# A Longitudinal Assessment of Associations between Adolescent Environment, Adversity Perception, and Economic Status on Fertility and Age of Menarche

**DOI:** 10.1371/journal.pone.0155883

**Published:** 2016-06-01

**Authors:** Dorsa Amir, Matthew R. Jordan, Richard G. Bribiescas

**Affiliations:** 1 Department of Anthropology, Yale University, New Haven, Connecticut, United States of America; 2 Department of Psychology, Yale University, New Haven, Connecticut, United States of America; London School of Hygiene and Tropical Medicine, UNITED KINGDOM

## Abstract

**Purpose:**

Perceptions of environmental adversity and access to economic resources in adolescence can theoretically affect the timing of life history transitions and investment in reproductive effort. Here we present evidence of correlations between variables associated with subjective extrinsic mortality, economic status, and reproductive effort in a nationally representative American population of young adults.

**Methods:**

We used a longitudinal database that sampled American participants (N ≥ 1,579) at four points during early adolescence and early adulthood to test whether perceptions of environmental adversity and early economic status were associated with reproductive effort.

**Results:**

We found that subjectively high ratings of environmental danger and low access to economic resources in adolescence were significantly associated with an earlier age of menarche in girls and earlier, more robust fertility in young adulthood.

**Conclusion:**

While energetics and somatic condition remain as possible sources of variation, the results of this study support the hypothesis that perceptions of adversity early in life and limited access to economic resources are associated with differences in reproductive effort and scheduling. How these factors may covary with energetics and somatic condition merits further investigation.

## 1. Introduction

Research across a multitude of disciplines has routinely underscored the importance of the early life environment in shaping later life outcomes. In particular, socioeconomic disadvantage and psychological adversity in early life appear to be highly influential and are associated with a suite of negative social and biological outcomes, such as greater exposure to violence [1]. Household income in early life is a strong measure of socioeconomic status and is often a powerful predictor of cognitive development and behavior in later life [2]. Less well-understood is environmental safety and the ways in which it can influence development. Often, the safety of the early environment is assessed through measures such as violent crime rates in the ZIP code of the area in which participants spent their childhoods [3]. Data at the ZIP code level is reasonable for inferring objective rates of crime, but it does not take into account individuals’ global judgments of their environment and within-region variation in subjective assessments of adversity. Subjective assessments may be particularly important on an individual level, as each person may be integrating both information about the external environment and information about the internal environment (i.e. health and somatic condition) to provide more accurate judgments about future states than objective rates alone, functioning as a ‘weather forecast’ for later life environments [4]. For that reason, here we focus on individuals’ global evaluations of the safety of their neighborhood, the likelihood of their future, and household income in early life. Using a longitudinal database, we explore how these early life variables may influence the timing and intensity of reproduction, utilizing life history theory (LHT) as a predictive framework.

### 1.1 Life History Theory

Life history theory (LHT), a concept rooted in evolutionary biology, is founded on the observation that the availability and allocation of resources are central to an organism’s fitness and often directly affect fecundity and mortality. As developmental trajectories are optimized according to environmental context, selection has shaped organisms that can be both specialized and flexible in their behavior [5]. This selection pressure can result in the evolution of phenotypic plasticity. Life history theory predicts that organisms exposed to high levels of mortality and limited resources will tend to have an earlier age of sexual maturity, larger broods, higher reproductive effort, and a shorter lifespan [6]. This constellation of traits is often referred to as a *fast life history*. Conversely, a *slow life history*, characterized by delayed maturity, smaller reproductive effort, and greater longevity, tends to occur in more stable environments with low rates of mortality and population density fluctuation [6].

### 1.2 Environmental stability

An important factor influencing the development and timing of life history traits is environmental stability, which can be captured in two main variables: *life expectancy* and *resource availability*. Adjustments in reproductive effort as reflected by age at reproductive maturation and fertility in response to experimentally induced variation in mortality has been documented in a number of species under various conditions, in which increased mortality tends to elevate reproductive effort [7,8] though a negative relationship has also been observed [9]. Human life history strategies are sensitive to these same cues from the environment. For example, individuals on the slower end of the life history spectrum tend to have fewer children later in life, invest more in parental effort, and generally live longer lives [10–12]. As these cues have existed throughout evolutionary history, selection pressure may have produced a psychology that uses this information to adaptively time life history stages in an effort to optimize fitness. A fast life history track may be a response produced via phenotypic plasticity to the specific ecology that poverty creates [13]. As such, data shows that individuals who are raised in neighborhoods suffering from socioeconomic deprivation often have an earlier and more robust reproductive output [13]. Further evidence suggests that county-specific crime rates significantly predict the age at which people had children [14]. These patterns also exist on a national level; across 170 nations, Low and colleagues found that 74% of the variation of age at first birth can be predicted by life expectancy [15]. Urban populations in high crime/high mortality areas also tend to exhibit evidence of greater reproductive effort through earlier ages of menarche and higher fertility [16]. Additionally, experimentally induced stress has been shown to decrease ideal female reproductive timing and this effect is particularly large in individuals who reported more exposure to childhood adversity [17].

### 1.3 Early life environment

An extended period of juvenile growth in humans allows for a longitudinal amalgamation of inputs from the environment, such as resource availability and environmental safety, in service of optimizing life history scheduling. These cues are potentially more important in pre-reproductive life as they can affect the onset, timing and intensity of reproduction. In line with this reasoning, previous research has shown that early life environment can have significant effects on later life outcomes [18–23], such as adolescent self-regulation [24], externalizing behavior [23], and risk taking and impulsivity [23]. While much of this research has focused on conditions in the first 5 to 7 years of life (i.e.[19]), it is also evident that conditions in adolescence play a role in the development of strategies [10,25]. Of particular interest to human biologists is reproductive output, which can be measured through variables such as age at reproductive maturation, age at first birth, and number of offspring. Reproductive maturation is more clearly captured in girls, rather than boys, by assessing the age at menarche. This life history event, while possessing a significant heritable component [26,27], is also importantly influenced by environmental factors such as psychosocial stressors [19,28]. In line with this argument, research has shown that factors such as the absence of a father [28], the quality of early family relationships [29], parent-child closeness [30], the presence of psychopathology [31], perception of family life environment [32], the presence of family conflict [33], food insecurity [34], and childhood sexual abuse [35] are all significantly associated with an earlier age at menarche. A thorough review of these factors in the development of human reproductive strategies can be found in a recent review by Jay Belsky [36].

### 1.4 Current study

The purpose of the current study is to assess associations between environmental and economic adversity in adolescence and early adult reproductive output and scheduling using a large, nationally-representative dataset. This dataset has previously been analyzed in a life history framework: in this paper, Brumbach and colleagues showed that both environmental harshness and unpredictability account for unique variation in the expression of adolescent and young adult life history strategy [10]. This study expands upon Brumbach et al.’s work in a number of ways. Firstly, while the authors also tested their hypotheses using the National Longitudinal Study of Adolescent Health, they only had access to three waves of data collection as the fourth wave had not been made available. Released in 2009, this new wave of data is a better measure of early fertility as it follows subjects to approximately the age of 34. As the mean age of the American mother at first birth is approximately 25 years [37], this most recent wave of data provides a more complete assessment of births in this cohort of adults. In addition, and most importantly, the Brumbach paper did not address age at menarche nor the timing of reproductive output. Therefore, we expand upon their work to consider the association between early life events and reproduction. We hypothesized that a greater global assessment of danger in the adolescent life environment would cue individuals to pursue a faster life history track, characterized by an earlier age of menarche and earlier, more robust fertility. In particular, this study focused on subjective measures of environmental safety to test how strongly perceptions of safety were associated with later life outcomes. As the nature of this study is associational, we cannot say with certainty the direction of causality; however, we believe the principles of life history theory and the results of studies similar to ours [10] can help illuminate the potential directionality of these associations, in which the local environment is the cause of changes in reproductive scheduling and output. Furthermore, while we could not address all the possible variables of influence on reproduction, we did have access to data regarding race, father absence, and adult income, which have been implicated as relevant factors to reproduction [38–40].

## 2. Materials and Methods

**Fig 1:**
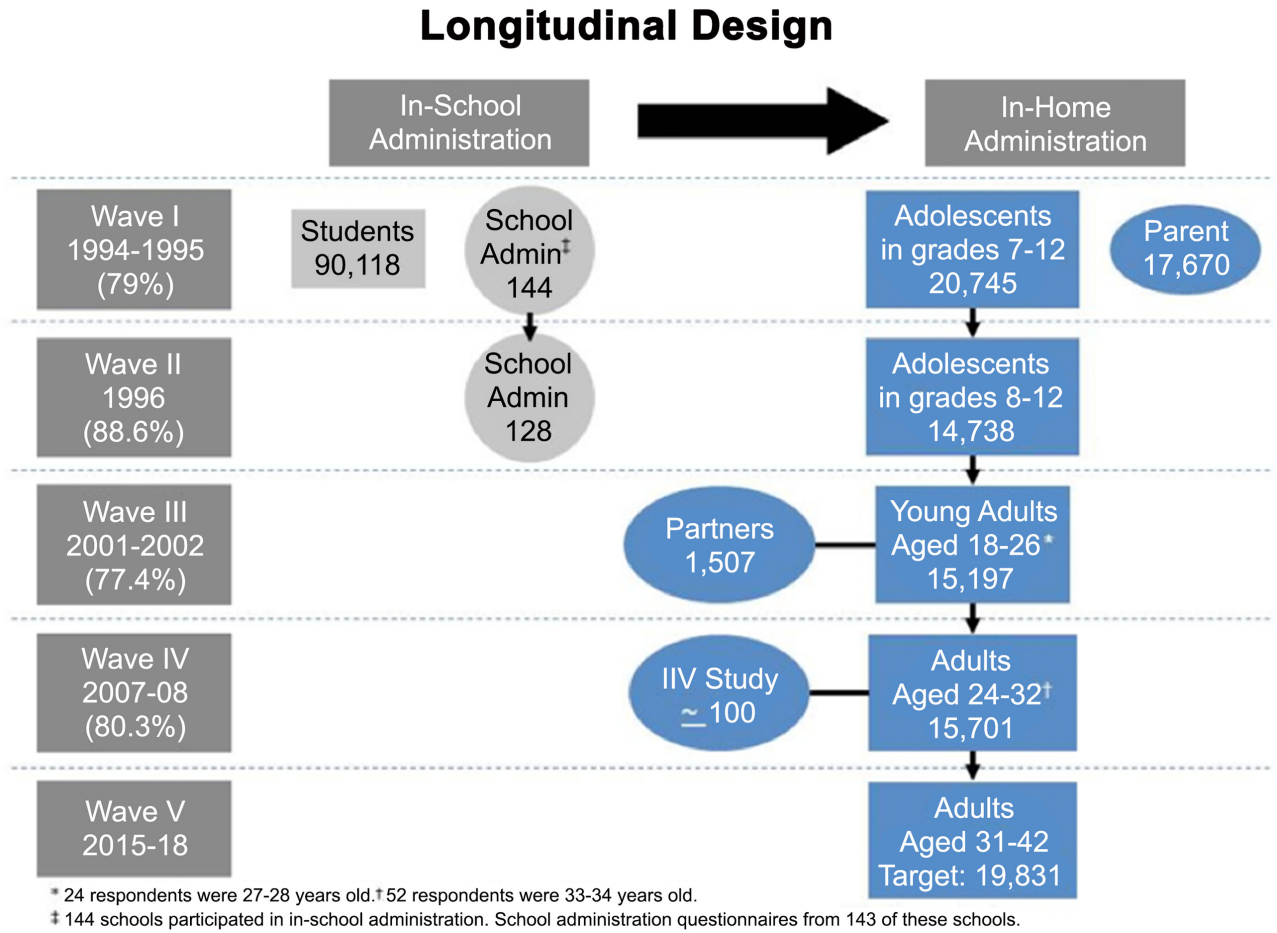
A schematic of the Add Health study design (reproduced from [33]).

In this study, we used data from the National Longitudinal Study of Adolescent Health (abbreviated as Add Health) to test our hypotheses [41]. This database is a nationally-representative sample of Americans with data being collected in four waves across 14 years. Wave 1 data collection occurred in 1994–1995 and targeted both male and female students in grades 7–12 (ages 12–21 in the subset who remained in the study through Wave IV; 90% between 13.3 and 18.7) across the United States [42]. Wave II and Wave III of data collection occurred in 1996 and 2001–2002, respectively, following the same individuals. Wave IV, the most recent wave, took place in 2007–2008 and collected data from the now-adults aged 24–34 (90% between 26.25 and 31.6). The goal of the Add Health study was to collect data for use in exploring the influences of both the individual attributes of respondents and the attitudes of their various environments on health and health-related behaviors [42]. A schematic of the study design is presented in Fig 1.

We used several variables from Wave I to synthesize a picture of the availability of resources and environmental safety in adolescence. We used family income as a proxy for early extra-somatic resource access. Perceived environmental safety was estimated with two Wave I variables: answers to the yes/no question “Do you feel safe in your neighborhood?” and an 8-point Likert scale question “On a scale of ‘No chance’ to ‘It will happen’, what do you think are the chances you will live to age 35?” (S1 Fig). Although we have no way of explicitly determining or accounting for extrinsic mortality rates, our predicting variables track individuals’ perceptions of adversity in their environments. As a check, we looked at the association between the number of times the individual witnessed a shooting/stabbing or was the victim of a shooting/stabbing. As expected, our two main variables assessing neighborhood safety ratings and predictions of early death were strongly correlated with the bodily harm questions, and thus can serve as effective summary variables for our purposes. Additionally, it’s possible the global judgments of safety may be capturing unexplained variance in the environment. While it would be ideal if we could simultaneously assess both objective and subjective measures of safety, the database does not offer individual-level objective safety rates. Therefore, despite the fact that actual risk and perceived risk may diverge [43], we used the perceived risk metrics to predict (a) number of live births up to age 34 among young adults of both sexes and (b) age at menarche in women. The data for menarche was obtained from Wave I, and the data for number of live births was obtained from Wave IV. Descriptive statistics of our sample are produced in Table 1.

**Table 1:** Descriptive statistics for the variables of interest.

**Sex**	**N**	
Male	1,428	
Female	2,426	
**Felt safe in neighborhood**	**N**	
Yes	3,342	
No	502	
**Lived with father**	**N**	
Yes	1,964	
No	748	
**Race**	**N**	
White	1,591	
Black	888	
Asian	101	
American Indian	176	
Other	201	
	**Mean**	**SD**
**Age**	28.72	1.7
**Likelihood of living to 35 (out of 9)**	6.53	1.99
**Age at menarche**	12.05	1.37
**Number of children**	1.78	1.21
**Early life income**	$40,376	$42,560
	**Median**	**Mode**
Adult income*	$57,500	$50,000–74,999

The descriptives above reflect values at the time of data collection in Wave 4 and are restricted to participants who were still present in the study during Wave 4.

*Adult income was reported during Wave 4 via income ranges, so determining a mean and standard deviation was impossible; therefore, the values above represent the median and mode.

## 3. Results

### 3.1 Age at menarche

**Fig 2:**
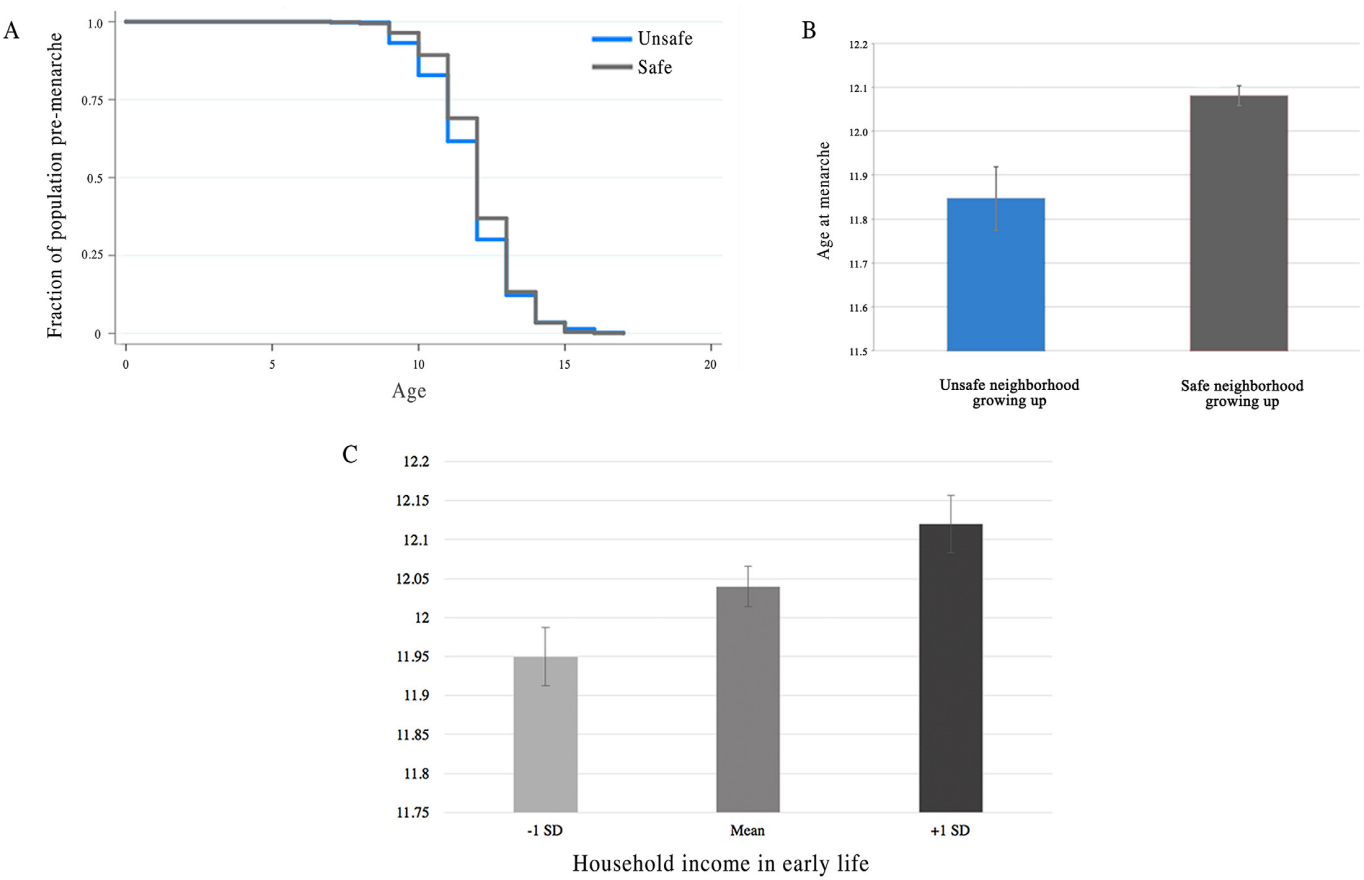
Correlates to age at menarche. **(A)** Kaplan-Meier survival estimation of percent of population who have not reached menarche by perceived safety of adolescent neighborhood. **(B)** Comparison of mean age at menarche by perceived safety of adolescent neighborhood, t(3,604) = 3.304, p = .0005. For both A & B graphics, N = 425 in unsafe neighborhood, N = 3,181 in safe neighborhood. **(C)** Comparison of age at menarche by household family income in early life, β = 0.0015, p<0.01, N = 2702.

We performed a Kaplan-Meier survival analysis to determine if women living in a neighborhood they assessed as unsafe in early life were more likely to have reached menarche earlier. As predicted by LHT, perceived safety of neighborhood is significantly associated with age at menarche (Fig 2). We performed a log-rank test for equality of survivor functions which revealed that individuals living in an unsafe neighborhood in early life were more likely to reach menarche at an earlier age than those who viewed their neighborhood as safe (χ^2^(1) = 6.09, p = 0.013).

We then used multiple linear regression analysis to determine which variables statistically predicted age at menarche (Table 2).

**Table 2:** Regression analysis of demographic variables predicting age at menarche.

Specification:	(1)	(2)	(3)	(4)	(5)	(6)	(7)
VARIABLES:							
**How likely to live to 35**	0.0487**					-0.00237	-0.00200
	(0.0149)					(0.0194)	(0.0199)
**Felt safe in neighborhood**		0.234***				0.194	0.146
		(0.0707)				(0.113)	(0.118)
**Individual income as an adult**			0.0219*			0.00870	0.00894
			(0.00913)			(0.0130)	(0.0133)
**Household income in early life**				0.00147**		0.00117*	0.00106*
				(0.000466)		(0.000526)	(0.000527)
**Lived with father in early life**					0.115	-0.0364	-0.0245
					(0.0591)	(0.0791)	(0.0816)
**Race (white omitted)**							
**Black**							-0.0386
							(0.0835)
**Asian**							-0.0537
							(0.210)
**American Indian**							-0.161
							(0.173)
**Other**							-0.233
							(0.128)
**Constant**	11.73****	11.85****	11.87****	11.97****	11.97****	11.77****	11.85****
	(0.103)	(0.0664)	(0.0740)	(0.0338)	(0.0505)	(0.176)	(0.192)
Observations	2,557	3,606	2,863	2,702	2,630	1,555	1,504
R-squared	0.004	0.003	0.002	0.004	0.001	0.006	0.008

Standard errors are in parentheses.

****p<0.0001,

*** p<0.001,

** p<0.01,

* p<0.05.

The predictions set forth by life history theory are supported by the data. We find that an individual’s perceived likelihood of living to the age of 35 (β = 0.049, p<0.001), whether or not they reported their neighborhood as safe (β = 0.234, p<0.001), and household income as a child (β = 0.0015, p<0.01) were all significant in the predicted direction (fewer resources and less early life safety are associated with an earlier age of menarche). The presence of a father was marginally significantly predictive (β = 0.115, p = 0.052). When all variables were included in the analysis, as in specification 6, household income in early life is the only robust predictor of age at menarche. However, we should note that the β-estimate of the effect of growing up in a safe neighborhood changes only slightly between specification two (the univariate estimate) and specification six (the multivariate estimate); this may be due in part to larger standard errors resulting from a smaller sample size (N = 3,606 vs. N = 1,555).

It is important to note that, in general, the average age of menarche occurs prior to Wave I sampling for most individuals. However, here we are interested in the *association* between the perception of environmental safety in adolescence and pubertal timing. Note that we are not predicting age at menarche in a chronological sense, but rather in a statistical sense. Additionally, as nearly 70% of individuals born into the bottom income quintile remain there for the rest of their lives [44], it is likely that perceptions of environmental conditions in adolescence are reflective of similar conditions in childhood. While there may be instances of environmental conditions changing rapidly in those few years, it is more likely that the environment is relatively stable and the information gathered in Wave I is reflective of the environment prior to menarche. In addition, when we control for the age at which individuals moved to the house they lived in during Wave I data collection, the analyses are unchanged.

### 3.2 Number of children in early adulthood

Female fertility (Table 3) and male fertility (Table 4) were analyzed separately.

**Table 3:** Female-specific regression analysis of demographic variables predicting number of live births in early adulthood.

Specification:	(1)	(2)	(3)	(4)	(5)	(6)	(7)
VARIABLES							
**Age**	0.0563***	0.0856****	0.0990****	0.0582***	0.0426*	0.0564**	0.0569**
	(0.0167)	(0.0142)	(0.0141)	(0.0163)	(0.0167)	(0.0195)	(0.0196)
**How likely to live to 35**	-0.0523***					-0.0196	-0.0126
	(0.0148)					(0.0182)	(0.0185)
**Felt safe in neighborhood**		-0.434****				-0.0555	-0.0877
		(0.0687)				(0.100)	(0.104)
**Individual income as an adult**			-0.119****			-0.0888****	-0.0925****
			(0.00846)			(0.0124)	(0.0127)
**Household income in early life**				-0.00350****		-0.00159*	-0.00150*
				(0.000579)		(0.000664)	(0.000662)
**Lived with father in early life**					-0.260****	-0.129	-0.164*
					(0.0607)	(0.0745)	(0.0760)
Race (white omitted):							
**Black**							-0.226**
							(0.0787)
**Asian**							-0.786***
							(0.219)
**American Indian**							-0.334*
							(0.169)
**Other**							0.0635
							(0.122)
	2.135****	2.241****	2.740****	1.978****	1.997****	2.799****	2.916****
Constant	(0.102)	(0.0636)	(0.0665)	(0.0362)	(0.0509)	(0.160)	(0.175)
Observations	1,689	2,419	2,300	1,776	1,747	1,173	1,133
R-squared	0.014	0.031	0.094	0.027	0.014	0.072	0.089

Standard errors are in parentheses.

**** p<0.0001,

*** p<0.001,

** p<0.01,

* p<0.05.

**Table 4:** Male-specific regression analysis of demographic variables predicting number of live births in early adulthood.

Specification:	(1)	(2)	(3)	(4)	(5)	(6)	(7)
VARIABLES							
**Age**	0.0774***	0.108****	0.121****	0.0898****	0.0875****	0.123****	0.128****
	(0.0219)	(0.0185)	(0.0191)	(0.0208)	(0.0217)	(0.0260)	(0.0268)
**How likely to live to 35**	-0.0468**					-0.0595**	-0.0677***
	(0.0165)					(0.0194)	(0.0198)
**Felt safe in neighborhood**		-0.492****				-0.548***	-0.515***
		(0.102)				(0.144)	(0.146)
**Individual income as an adult**			-0.0808****			-0.0489**	-0.0503**
			(0.0127)			(0.0180)	(0.0182)
**Household income in early life**				-0.00524****		-0.00349**	-0.00316*
				(0.00107)		(0.00133)	(0.00132)
**Lived with father in early life**					-0.0647	0.0853	0.113
					(0.0848)	(0.104)	(0.108)
Race (white omitted):							
**Black**							0.269*
							(0.106)
**Asian**							0.165
							(0.232)
**American Indian**							0.411*
							(0.160)
**Other**							0.720****
							(0.184)
	1.801****	2.017****	2.228****	1.757****	1.556****	2.862****	2.710****
Constant	(0.112)	(0.0959)	(0.107)	(0.0557)	(0.0742)	(0.228)	(0.239)
Observations	946	1,425	1,324	1,081	965	676	650
R-squared	0.021	0.037	0.054	0.036	0.017	0.086	0.119

Standard errors are in parentheses.

**** p<0.0001,

*** p<0.001,

** p<0.01,

* p<0.05.

We used multiple linear regression analysis to determine which variables statistically predict the total number of live births as young adults. Since we do not have complete fertility data, this is a measure of reproductive scheduling, assessing whether individuals have more children at an earlier age. LHT predicts that adolescent environments marked by resource scarcity and low environmental safety may induce faster life history tracks with earlier and more robust reproduction. Indeed, we find this pattern in the data.

Among women, an individual’s perceived likelihood of living to the age of 35 (β = -0.0523, p<0.001), whether or not they reported their neighborhood as safe (β = -0.434, p<0.0001), and family income in early life (β = -0.00350, p<0.0001) were all significant in the predicted direction (fewer resources and less environmental safety are associated with more children). Living with one’s father in early life was also negatively predictive (β = -0.260, p<0.0001) of the number of children, in line with similar research showing that father absence is associated with early sexual activity and teenage pregnancy [39]. Individual income as an adult was also negatively predictive of fertility (β = -0.119, p<0.0001). All of these variables are individually predictive, but in order to ensure that these variables were predicting different variance, we built specifications into the model in Table 3. When all the variables are present in the same statistical model, household income in early life remains predictive of female fertility (β = -0.00150, p<0.05), as does individual income as an adult (β = -0.0925, p<0.0001), and father presence (β = -0.164, p<0.05). Race appears to play a role in fertility as well, as the racial categories of “Black” (β = -0.226, p<0.01), “Asian” (β = -0.786, p<0.001), and “American Indian” (β = -0.334, p<0.05) are associated with a lower of number of live births, as relative to those identifying as “White”. The use of birth control is also an important variable when discussing fertility; however, due to the fact that the majority of participants did not provide birth control information, this variable has not been included in the primary analysis; you can find more information about this in the supplementary materials (S1 Table). We also analyzed one possible mediating variable in the relationship between living in a neighborhood that was judged as safe and the fertility: the use of contraceptives during recent sexual encounters (S1 File).

Among men, a greater number of early life variables were associated with later life fertility. We replicate the effects found on female fertility, such that an individual’s perceived likelihood of living to the age of 35 (β = -0.0468, p<0.01), whether or not they reported their neighborhood as safe (β = -0.492, p<0.0001), individual income as an adult ((β = -0.0808, p<0.0001), and family income in early life (β = -0.00524, p<0.0001) were all significant in the predicted direction (fewer resources and less environmental safety are associated with more children). Interestingly with the men, however, when all variables are included in the model, almost all remain significant, such that the likelihood of living to age 35 (β = -0.0677, p<0.001), ratings of environmental safety (β = -0.515, p<0.001), individual income as an adult (β = -0.0503, p<0.01), and household income in early life (β = -0.00316, p<0.05) all have some bearing on male fertility. Race also plays a role, such that participants who identify as “Black” (β = 0.269, p<0.05) and “Other” (β = 0.720, p<0.0001) tend to have higher fertility relative to those identifying as “White”.

**Fig 3:**
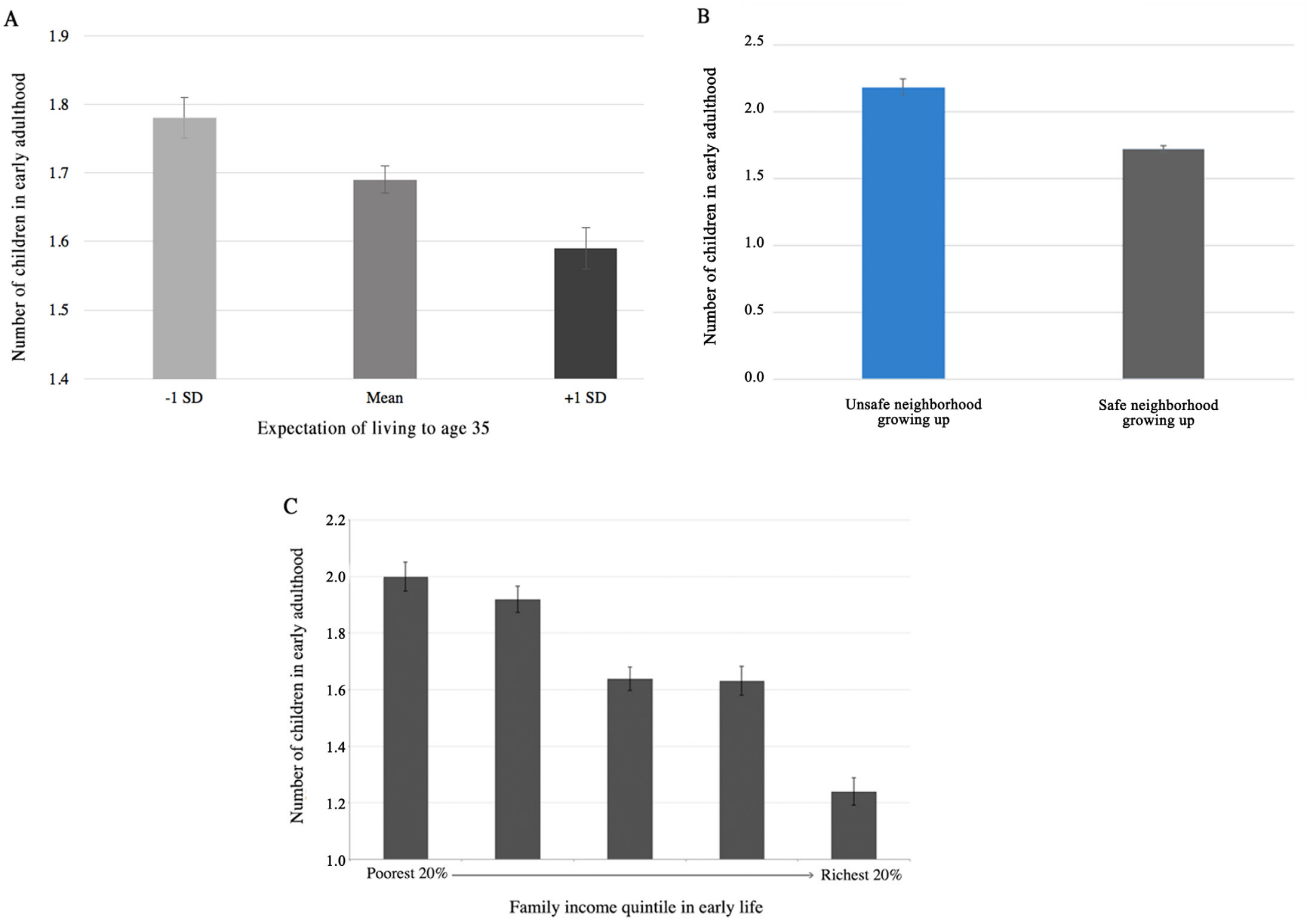
Correlates to number of children in early adulthood. **(A)** Comparison of number of children in early adulthood by expectation of living to age 35 reported as mean, +1 standard deviation from the mean, and -1 standard deviation from the mean, β = -0.046, p<0.0001, N = 2,365. (B) Number of children in early adulthood by perceived safety of early environment, β = -0.467, p<0.0001, N = 3,844. **(C)** Number of children in early adulthood by family income quintile in adolescence, β = -0.004, p<0.0001, N = 2,859.

When we combine both sexes, all three early life variables of interest—perceived likelihood of living to age 35, neighborhood safety ratings, and household income in early life—remain important in predicting fertility (Fig 3).

## 4. Discussion

Our results support the hypothesis that early economic access and global assessments of environmental adversity are associated with variation in reproductive effort in a contemporary human population. In the case of both menarche and reproductive scheduling, we find that lower access to resources and lower ratings of environmental safety in adolescence are associated with an earlier age at menarche in girls and a larger quantity of offspring at earlier ages in both sexes. Our findings are consistent with predictions made by life history theory in regards to presence and perception of available resources and extrinsic hazards. These results support the socialization model proposed by Belsky and colleagues [19], in which an early environment marked by low access to resources (i.e., household income) and perceived danger is correlated with a greater number of children and an earlier age at menarche. While other studies have examined extrinsic mortality on reproductive effort in women [45], as well as deployed the use of longitudinal data to assess other aspects of human life history biology [46–49], the availability and use of a longitudinal dataset to assess environmental adversity and reproductive effort in a human population is rare.

Through our analyses, we were able to identify some variables that are more predictive than others when considered in a larger model. While judgments of environmental safety are relevant in shaping reproduction, it appears that household income in early life may be exerting a larger influence. When compared to our other variables of interest, early experience of economic adversity appears to be the strongest predictor of age at menarche in women an important predictor of fertility, as well. This could be because access to economic resources covaries with many other determinants of life outcomes, such as quality of education, access to medical resources, and more. There is a growing body of literature emphasizing the impact of income inequality on life outcomes, and given the rise of economic inequality in the United States [50], this finding may help us understand the biological and demographic consequences of these economic transitions. In addition, findings like the ones presented here underscore the importance of economic resources in early life and may help guide us toward more effective public health and policy interventions.

Our results further highlight differential effects of the early life environment on male and female fertility. Among both sexes, we find that low household income in early life, low individual income as adults, perceptions of early environmental danger, and perceptions of high extrinsic mortality are correlated with more births in early adulthood. When incorporating all of these variables in a model, however, sex differences emerge. While both household income in early life and individual income as an adult appear to play a role in fertility patterns in this model, it appears that male fertility remains strongly correlated with perceptions of early environmental safety, while female fertility does not. One possible explanation is that males are more sensitive to information regarding extrinsic mortality and safety in an environment, given that males are differentially involved in violence, both as perpetrators and victims [51]. These differences among the sexes merit further investigation.

While this study is demographic in nature, the variables of reproductive effort require the identification of potential biological pivot points that should contribute to variation in age of menarche and fertility. Responses to assessments of environmental adversity and psychosocial stress are likely to involve the hypothalamic-pituitary-adrenal (HPA) axis which plays an important role in reproductive maturation, inter-birth intervals, and fertility, as perceptions of environmental adversity are known to influence adrenal function and the production of glucocorticoids [52]. Adrenal activation commonly involves increases in the secretion of adrenal androgens that can contribute to the desensitization of target receptors in the hypothalamus, thereby facilitating the onset of menarche. Additionally, obesity and metabolic syndrome, which are disproportionately present in communities with environmental adversity, are contextually important in the present investigation since these disorders are increasingly common and have been linked with accelerated reproductive maturation [53]. Obese children and adolescents are exposed to higher levels of ovarian androgens that may also contribute to earlier age of menarche [54–56]. Clearly, associations between the high and growing incidence of obesity and perceptions of environmental adversity may covary and merit further investigation [57,58]. Environmental adversity may affect early adult fertility mechanistically through decreases in interbirth intervals which may include increases in ovarian function (higher estrogen levels)[59], shorter periods of lactational amenorrhea [60], increased coital activity, or shorter gestational periods. While the role of energetics and metabolic effects on hormonal factors are crucial for the timing of menarche, this does not obviate the role of global assessments of environmental risk. Indeed, during acute or chronic stress, the activation of the hypothalamic-pituitary-adrenal axis is, at its core, a metabolic response to increase glucose availability and facilitate the utilization of energetic resources to deal with environmental risks and challenges. Among girls who endured extremely stressful wartime conditions, age of menarche increased, presumably due to the high stress conditions; however, investigations such as this are confounded by the energetic and nutritional stresses that are likely to also be common in wartime situations [61]. For example among Ugandan girls, wartime trauma and stress was not associated with age of menarche although better nutritional status did correlate with significantly lower ages of menarche [62]. In addition, while socioeconomic status in general has been found to covary with age at menarche (such as in [63]), this result is not necessarily unanimous (see review in [64]) and may be less important in developed countries when compared to developing countries [65]. However, our results suggest that socioeconomic status remains important, even in a developed country like the United States.

While the results are supportive of our central hypothesis and provide novel information on the human life history, there are important constraints and limitations to our study that need to be considered. First, despite the unique and informative nature of using a longitudinal database, the influence of other factors on menarche and fertility, most importantly somatic condition, physical activity, and the effects of caloric intake on age of menarche and adult fecundity could not be addressed. It is well-documented that caloric availability and energetic status are positively associated with age of menarche [66]. In addition, energy balance as determined by physical activity, exercise, and caloric intake all have significant influences on ovarian function, fecundity, and reproductive output [67,68]. Indeed, it has been suggested that developmental conditions during childhood and adolescence can have downstream effects on adult ovarian function and fertility [69,70]. Therefore, further investigation of how these energetic variables may influence and interface with with judgments of safety and access to resources should be undertaken. Secondly, it is likely that psychosocial stress covaries with energetic factors in several important ways, such that increased reproductive effort in the face of environmental adversity is more likely to occur when caloric availability is sufficient or even high, which is common in low SES communities in the United States. Thirdly, as these data are derived from observation of naturally occurring trends, they cannot be used to definitively determine causation. And lastly, these data are also dependent on self-reported responses, and are subject to common problems associated with this form of report.

In summary, this investigation outlines associations between judgments of environmental safety, early access to economic resources, and increases in reproductive effort. While the role of other factors that are common to high adversity environments remain to be fully elucidated, it is becoming increasingly clear that the timing of key life history events such as menarche and variation in fertility among humans is subject to similar stresses and influences as other organisms. Our findings suggest that life history strategies may be influenced by adolescent environments and have important consequences on reproductive outcomes. It is also likely that other areas of adult behavior beyond reproduction are affected as well, in more cognitive domains such as risk preferences and mate choice. These results are important in highlighting the utility and effectiveness of life history theory in analyzing human behavior—even for populations of humans living in environments radically different than those in their evolutionary histories, like those living in the United States. We believe the deployment of biodemographic methods for assessing human evolutionary biology is a fruitful method of advancing our knowledge of the evolution of our species as well as providing much needed insights into the health and social conditions of underserved communities.

## Supporting Information

S1 Fig Distribution of answers to the question “On a scale of ‘No chance’ to ‘It will happen’, what do you think are the chances you will live to age 35?”. N = 2635.
(PNG)

S1 File Mediation Analysis. (DOCX)

S1 Table Multiple regression analysis that has been extended from the main text to include the use of birth control early in life. There was very little overlap between birth control usage and the other variables, but we include birth control in these analyses for those who are interested. Standard errors are in parentheses. ****p<0.0001, *** p<0.001, ** p<0.01, * p<0.05.
(DOCX)
