# Supplementary figures and images for: A Longitudinal Assessment of Associations between Adolescent Environment, Adversity Perception, and Economic Status on Fertility and Age of Menarche
Source: PLoS One. 2016 Jun 1;11(6):e0155883. doi: 10.1371/journal.pone.0155883 (PMC4889152; doi:10.1371/journal.pone.0155883)

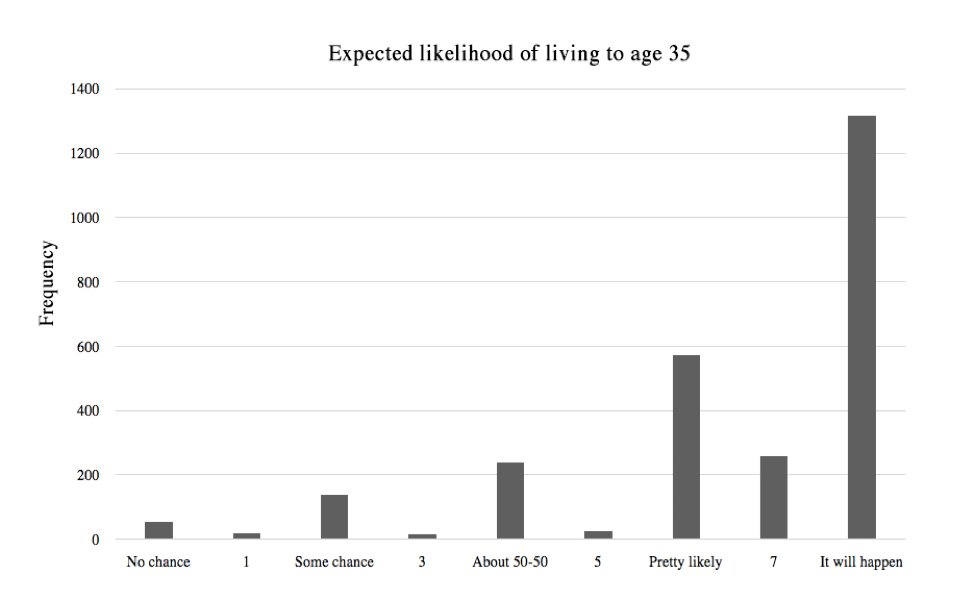

Supplement: S1 Fig — N = 2635. (PNG) [file pone.0155883.s001.png]
