# Supplementary material for: A Longitudinal Assessment of Associations between Adolescent Environment, Adversity Perception, and Economic Status on Fertility and Age of Menarche
Source: PLoS One. 2016 Jun 1;11(6):e0155883. doi: 10.1371/journal.pone.0155883 (PMC4889152; doi:10.1371/journal.pone.0155883)
Supplement: S1 File — (DOCX) [file pone.0155883.s002.docx]

**S1 File. Mediation Analysis.**
We were able to look at one possible mediating variable in the relationship between living in a neighborhood that was judged as safe and the fertility: the use of contraceptives during recent sexual encounters. That is, we tested the hypothesis that part of the reason that those who grew up in neighborhood they judged to be unsafe went on to have more children is that those who judged their childhood environments to be unsafe were less likely to use contraception. To test this hypothesis, we conducted a mediation analysis. We began the mediation analysis by assessing the direct effect of growing up in an unsafe neighborhood on fertility and found a significant relationship, b=-0.610, SE=0.081, p<0.001, such that neighborhood safety was associated with fewer children. We next assessed the relationship between childhood neighborhood safety assessments and use of a contraceptive during the last sexual encounter the respondents had, and found a significant relationship, b=0.073, SE=0.031, p=0.017, such that growing up in a safe neighborhood was associated with more contraceptive use. When we controlled for contraceptive use, b=-0.271, SE=0.058, p<0.001, we saw a small decrease in the relationship between childhood neighborhood safety and fertility, b=-0.590, SE=0.080, p<0.001, which did result in a significant partial mediation, b=-0.020, Sobel’s z=-2.121, p=0.03.
