# Supplementary material for: A Longitudinal Assessment of Associations between Adolescent Environment, Adversity Perception, and Economic Status on Fertility and Age of Menarche
Source: PLoS One. 2016 Jun 1;11(6):e0155883. doi: 10.1371/journal.pone.0155883 (PMC4889152; doi:10.1371/journal.pone.0155883)
Supplement: S1 Table — There was very little overlap between birth control usage and the other variables, but we include birth control in these analyses for those who are interested. Standard errors are in parentheses. ****p<0.0001, *** p<0.001, ** p<0.01, * p<0.05. (DOCX) [file pone.0155883.s003.docx]

| Specification: | (1) | (2) | (3) | (4) | (5) | | (6) | (7) | (8) | (9) | | (10) |  |
| --- | --- | --- | --- | --- | --- | --- | --- | --- | --- | --- | --- | --- | --- |
| **VARIABLES** |  | | | | | | | | | | | | |
|  |  |  |  |  | |  |  |  |  | |  |  |  |
| **Age** | 0.0563*** | 0.0856**** | 0.0990**** | 0.0582*** | | 0.0426* | 0.0564** | 0.0569** | 0.0413 | | 0.0206 | 0.0214 |  |
|  | (0.0167) | (0.0142) | (0.0141) | (0.0163) | | (0.0167) | (0.0195) | (0.0196) | (0.0485) | | (0.0636) | (0.0634) |  |
| **How likely to live to 35** | -0.0523*** |  |  |  | |  | -0.0196 | -0.0126 |  | | 0.0456 | 0.0291 |  |
|  | (0.0148) |  |  |  | |  | (0.0182) | (0.0185) |  | | (0.0505) | (0.0506) |  |
| **Felt safe in neighborhood** |  | -0.434**** |  |  | |  | -0.0555 | -0.0877 |  | | 0.143 | 0.123 |  |
|  |  | (0.0687) |  |  | |  | (0.100) | (0.104) |  | | (0.229) | (0.237) |  |
| **Individual income as an adult** |  |  | -0.119**** |  | |  | -0.0888**** | -0.0925**** |  | | -0.0245 | -0.00123 |  |
|  |  |  | (0.00846) |  | |  | (0.0124) | (0.0127) |  | | (0.0329) | (0.0337) |  |
| **Household income in early life** |  |  |  | -0.00350**** | |  | -0.00159* | -0.00150* |  | | -0.0132**** | -0.0123*** |  |
|  |  |  |  | (0.000579) | |  | (0.000664) | (0.000662) |  | | (0.00320) | (0.00315) |  |
| **Lived with father in early life** |  |  |  |  | | -0.260**** | -0.129 | -0.164* |  | | -0.0269 | 0.0872 |  |
|  |  |  |  |  | | (0.0607) | (0.0745) | (0.0760) |  | | (0.185) | (0.190) |  |
| **Taking birth control in early life** |  |  |  |  | |  |  |  | -0.413*** | | -0.105 | -0.126 |  |
|  |  |  |  |  | |  |  |  | (0.116) | | (0.166) | (0.166) |  |
| **Race (white omitted)** |  |  |  |  | |  |  |  |  | |  |  |  |
| **Black** |  |  |  |  | |  |  | -0.226** |  | |  | 0.103 |  |
|  |  |  |  |  | |  |  | (0.0787) |  | |  | (0.224) |  |
| **Asian** |  |  |  |  | |  |  | -0.786*** |  | |  | -1.898** |  |
|  |  |  |  |  | |  |  | (0.219) |  | |  | (0.576) |  |
| **American Indian** |  |  |  |  | |  |  | -0.334* |  | |  | -0.564 |  |
|  |  |  |  |  | |  |  | (0.169) |  | |  | (0.408) |  |
| **Other** |  |  |  |  | |  |  | 0.0635 |  | |  | -0.280 |  |
|  |  |  |  |  | |  |  | (0.122) |  | |  | (0.264) |  |
|  | 2.135**** | 2.241**** | 2.740**** | 1.978**** | | 1.997**** | 2.799**** | 2.916**** | 2.445**** | | 2.428**** | 2.370**** |  |
| Constant | (0.102) | (0.0636) | (0.0665) | (0.0362) | | (0.0509) | (0.160) | (0.175) | (0.106) | | (0.385) | (0.410) |  |
|  |  |  |  |  | |  |  |  |  | |  |  |  |
| Observations | 1,689 | 2,419 | 2,300 | 1,776 | | 1,747 | 1,173 | 1,133 | 495 | | 225 | 220 |  |
| R-squared | 0.014 | 0.031 | 0.094 | 0.027 | | 0.014 | 0.072 | 0.089 | 0.027 | | 0.110 | 0.165 |  |

**S1 Table**
